# Diaqua­bis(5-carb­oxy-2-methyl-1*H*-imidazole-4-carboxyl­ato-κ^2^ *N* ^3^,*O* ^4^)cobalt(II) dimethyl­formamide disolvate

**DOI:** 10.1107/S1600536809012902

**Published:** 2009-04-10

**Authors:** Si-Ping Tang

**Affiliations:** a Department of Chemistry and Material Science, Hengyang Normal University, Hengyang, Hunan 421008, People’s Republic of China.

## Abstract

In the title compound, [Co(C_6_H_5_N_2_O_4_)_2_(H_2_O)_2_]·2C_3_H_7_NO, the Co^II^ ion lies on an inversion center and adopts a slightly distorted CoN_2_O_4_ octa­hedral geometry binding two bidentate chelating 5-carb­oxy-2-methyl-1*H*-imidazole-4-carboxyl­ate (H_2_MIDA^−^) monoanionic ligands and two axial aqua ligands. In the crystal structure, inter­molecular O—H⋯O hydrogen bonds link neighboring mol­ecules, generating a two-dimensional framework containing eight-membered H_4_O_4_ rings. In addition, the dimethyl­formamide solvent mol­ecules are hydrogen bonded to the two-dimensional framework *via* the NH groups of the H_2_MIDA^−^ ligands.

## Related literature

For background to *N*-heterocyclic carboxylic acids as ligands in coordination complexes, see: Gao *et al.* (2004 ▶); Shimizu *et al.* (2004 ▶); Zhang *et al.* (2006 ▶). For related structures, see: Liu *et al.* (2007 ▶); Nie *et al.* (2007 ▶); Zeng *et al.* (2008 ▶).
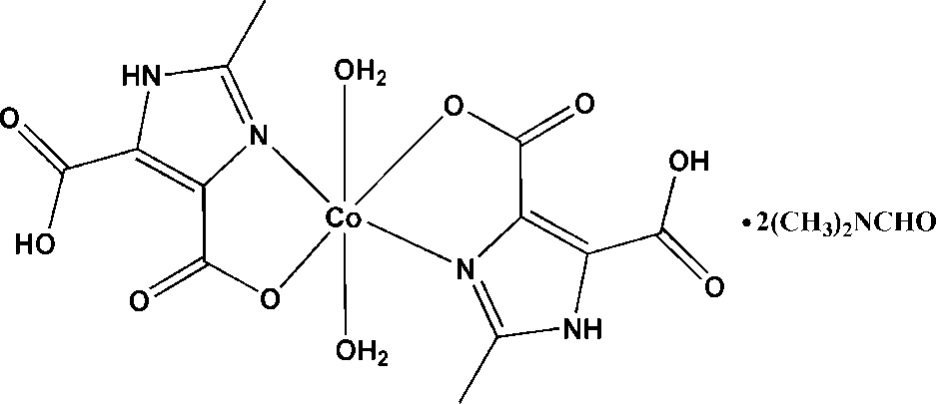


## Experimental

### 

#### Crystal data

[Co(C_6_H_5_N_2_O_4_)_2_(H_2_O)_2_]·2C_3_H_7_NO
*M* *_r_* = 579.39
Triclinic, 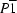

*a* = 7.1979 (11) Å
*b* = 9.2180 (15) Å
*c* = 10.8659 (17) Å
α = 65.173 (2)°
β = 83.459 (2)°
γ = 68.254 (2)°
*V* = 607.02 (17) Å^3^
*Z* = 1
Mo *K*α radiation
μ = 0.78 mm^−1^
*T* = 123 K
0.20 × 0.15 × 0.14 mm

#### Data collection

Bruker SMART APEX area-detector diffractometer
Absorption correction: multi-scan (*SADABS*; Sheldrick, 1996 ▶) *T*_min_ = 0.859, *T*_max_ = 0.899
4588 measured reflections
2335 independent reflections
2176 reflections with *I* > 2σ(*I*)
*R*_int_ = 0.026

#### Refinement

*R*[*F* ^2^ > 2σ(*F* ^2^)] = 0.039
*wR*(*F* ^2^) = 0.106
*S* = 1.06
2335 reflections
172 parameters
1 restraint
H atoms treated by a mixture of independent and constrained refinement
Δρ_max_ = 0.65 e Å^−3^
Δρ_min_ = −0.49 e Å^−3^

### 

Data collection: *SMART* (Bruker, 2002 ▶); cell refinement: *SAINT* (Bruker, 2002 ▶); data reduction: *SAINT*; program(s) used to solve structure: *SHELXS97* (Sheldrick, 2008 ▶); program(s) used to refine structure: *SHELXL97* (Sheldrick, 2008 ▶); molecular graphics: *SHELXTL* (Sheldrick, 2008 ▶); software used to prepare material for publication: *SHELXTL*.

## Supplementary Material

Crystal structure: contains datablocks I, global. DOI: 10.1107/S1600536809012902/sj2611sup1.cif

Structure factors: contains datablocks I. DOI: 10.1107/S1600536809012902/sj2611Isup2.hkl

Additional supplementary materials:  crystallographic information; 3D view; checkCIF report

## Figures and Tables

**Table 1:** Hydrogen-bond geometry (Å, °)

*D*—H⋯*A*	*D*—H	H⋯*A*	*D*⋯*A*	*D*—H⋯*A*
N2—H2⋯O5^i^	0.86	1.83	2.670 (2)	166
O2—H2*A*⋯O3	0.863 (10)	1.593 (11)	2.452 (2)	173 (3)
O1*W*—H1*W*2⋯O1^ii^	0.85	1.91	2.7579 (19)	178
O1*W*—H1*W*1⋯O1^iii^	0.85	2.03	2.847 (2)	160

Symmetry codes: (i) 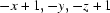; (ii) 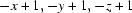; (iii) 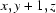.

**Fig. 1.**
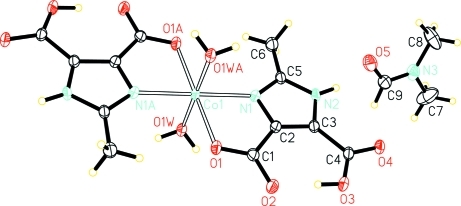
The title molecule with displacement ellipsoids drawn at the 50% probability level, and with the H atoms shown as spheres of arbitrary radii.

**Fig. 2.**
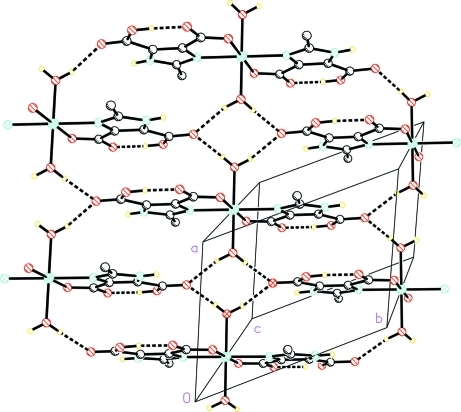
Packing diagram of the title compound showing the intra/intermolecular O—H···O hydrogen bonds as dashed lines. The H atoms not involved in hydrogen bonds and the DMF solvate molecules have been omitted for clarity.

## supplementary crystallographic information

### Comment

N-heterocyclic carboxylic acids have attracted considerable interests as ligands in metal complexes because of their structural diversity as multidentate chelating or bridging ligands (Gao *et al.*, 2004; Shimizu *et al.*, 2004; Zhang *et al.*, 2006). Recently, 2-methyl-1*H*-imidazole-4,5- dicarboxylic acid (H_3_MIDA) has been used as a chelating ligand to generate mononuclear complexes with cadmium (II), cobalt (II) and manganese (II) ions (Liu *et al.*, 2007; Nie *et al.*, 2007; Zeng *et al.*, 2008). We report here the synthesis and structure of a new cobalt (II) complex incorporating H_3_MIDA.

The title compound is composed of one Co (II) ion, two mono-deprotonated H_2_MIDA ligands, two aqua ligands and two DMF solvent molecules, Fig 1. The Co (II) cation lies on a crystallographic inversion center and has a distorted octahedral geometry with the basal plane occupied by two carboxylate O atoms and two N atoms from two chelating H_2_MIDA^-^ ligands. There are two axial aqua ligands. In the H_2_MIDA ligand, the carboxyl and carboxylate groups form an intramolecular hydrogen bond with an O···O distance of 2.452 (2) Å.

In the crystal packing, each aqua ligand is involved in two intermolecular O—H···O hydrogen bonds with two carboxyl O atoms from two neighboring molecules to genetrate a two-dimensional supramolecular structure (Fig. 2), in which two aqua ligands and two carboxyl O atoms form a H_4_O_4_ eight-membered ring. In addition, the DMF solvates are hydrogen-bonded to the two-dimensional framework *via* the –NH groups of the H_2_MIDA^-^ ligands.

### Experimental

A solution of cobalt perchlorate hexahydrate (73.2 mg, 0.2 mmol) and H_3_MIDA (15.8 mg, 0.1 mmol) in DMF (6 ml) and methanol (1 ml) was stirred for 5 h. After filtering, the filtrate was left for about two months and pink, block-like crystals of the title compound appeared. Yield: 11 mg (38%).

### Refinement

The carboxyl and water H atoms were located in a difference Fourier map and refined with *U*_iso_=1.5*U*_eq_ (O). The O—H distances of water were refined with idealized values of 0.85 Å, however, that of carboxyl is refined freely. All other H-atoms were positioned geometrically and refined using a riding model with C—H (methyl) = 0.96 Å, C—H (aldehyde) = 0.93 Å, N—H = 0.86 Å, and *U*_iso_ = 1.2*U*_eq_ (C, N).

### Crystal data

**Table:**

[Co(C_6_H_5_N_2_O_4_)_2_(H_2_O)_2_]·2C_3_H_7_NO	*Z* = 1
*M**_r_* = 579.39	*F*(000) = 301
Triclinic, *P*1	*D*_x_ = 1.585 Mg m^−^^3^
Hall symbol: -P 1	Mo *K*α radiation, λ = 0.71073 Å
*a* = 7.1979 (11) Å	Cell parameters from 3423 reflections
*b* = 9.2180 (15) Å	θ = 2.6–27.8°
*c* = 10.8659 (17) Å	µ = 0.78 mm^−^^1^
α = 65.173 (2)°	*T* = 123 K
β = 83.459 (2)°	Block, pink
γ = 68.254 (2)°	0.20 × 0.15 × 0.14 mm
*V* = 607.02 (17) Å^3^	

### Data collection

**Table:**

Bruker SMART APEX area-detector diffractometer	2335 independent reflections
Radiation source: fine-focus sealed tube	2176 reflections with *I* > 2σ(*I*)
graphite	*R*_int_ = 0.026
φ and ω scans	θ_max_ = 26.0°, θ_min_ = 2.6°
Absorption correction: multi-scan (*SADABS*; Sheldrick, 1996)	*h* = −8→7
*T*_min_ = 0.859, *T*_max_ = 0.899	*k* = −11→11
4588 measured reflections	*l* = −13→13

### Refinement

**Table:**

Refinement on *F*^2^	Primary atom site location: structure-invariant direct methods
Least-squares matrix: full	Secondary atom site location: difference Fourier map
*R*[*F*^2^ > 2σ(*F*^2^)] = 0.039	Hydrogen site location: inferred from neighbouring sites
*wR*(*F*^2^) = 0.106	H atoms treated by a mixture of independent and constrained refinement
*S* = 1.06	*w* = 1/[σ^2^(*F*_o_^2^) + (0.0631*P*)^2^ + 0.2477*P*] where *P* = (*F*_o_^2^ + 2*F*_c_^2^)/3
2335 reflections	(Δ/σ)_max_ < 0.001
172 parameters	Δρ_max_ = 0.65 e Å^−^^3^
1 restraint	Δρ_min_ = −0.49 e Å^−^^3^

### Special details

**Table:**

Geometry. All e.s.d.'s (except the e.s.d. in the dihedral angle between two l.s. planes) are estimated using the full covariance matrix. The cell e.s.d.'s are taken into account individually in the estimation of e.s.d.'s in distances, angles and torsion angles; correlations between e.s.d.'s in cell parameters are only used when they are defined by crystal symmetry. An approximate (isotropic) treatment of cell e.s.d.'s is used for estimating e.s.d.'s involving l.s. planes.
Refinement. Refinement of *F*^2^ against ALL reflections. The weighted *R*-factor *wR* and goodness of fit *S* are based on *F*^2^, conventional *R*-factors *R* are based on *F*, with *F* set to zero for negative *F*^2^. The threshold expression of *F*^2^ > σ(*F*^2^) is used only for calculating *R*-factors(gt) *etc*. and is not relevant to the choice of reflections for refinement. *R*-factors based on *F*^2^ are statistically about twice as large as those based on *F*, and *R*- factors based on ALL data will be even larger.

### Fractional atomic coordinates and isotropic or equivalent isotropic displacement parameters (Å^2^)

**Table:**

	*x*	*y*	*z*	*U*_iso_*/*U*_eq_	
Co1	0.0000	1.0000	0.5000	0.01688 (15)	
O4	0.1583 (2)	0.97632 (18)	0.32480 (14)	0.0200 (3)	
O1W	0.2666 (2)	0.96647 (18)	0.58550 (14)	0.0214 (3)	
H1W2	0.3716	0.9017	0.5643	0.032*	
H1W1	0.2857	1.0585	0.5685	0.032*	
O3	0.3261 (2)	0.78687 (18)	0.23787 (14)	0.0220 (3)	
O2	0.4205 (2)	0.47831 (19)	0.30617 (15)	0.0233 (3)	
H2A	0.391 (4)	0.5850 (15)	0.288 (3)	0.035*	
O1	0.3990 (2)	0.24920 (18)	0.48355 (16)	0.0241 (3)	
O5	0.8297 (3)	−0.1528 (2)	0.20333 (17)	0.0343 (4)	
N1	0.0922 (2)	0.7355 (2)	0.55599 (16)	0.0171 (4)	
N2	0.1798 (2)	0.4572 (2)	0.62169 (17)	0.0181 (4)	
H2	0.1932	0.3523	0.6713	0.022*	
N3	0.6518 (3)	0.1293 (2)	0.08740 (18)	0.0296 (4)	
C6	0.2260 (3)	0.8282 (3)	0.33158 (19)	0.0179 (4)	
C5	0.1936 (3)	0.6909 (2)	0.4550 (2)	0.0174 (4)	
C4	0.2493 (3)	0.5176 (2)	0.4945 (2)	0.0173 (4)	
C3	0.3636 (3)	0.4049 (3)	0.4250 (2)	0.0194 (4)	
C2	0.0862 (3)	0.5915 (2)	0.6556 (2)	0.0181 (4)	
C1	−0.0105 (3)	0.5776 (3)	0.7860 (2)	0.0247 (5)	
H1A	0.0882	0.5048	0.8597	0.037*	
H1B	−0.1121	0.5297	0.7961	0.037*	
H1C	−0.0703	0.6892	0.7866	0.037*	
C7	0.4556 (5)	0.2630 (4)	0.0431 (3)	0.0559 (8)	
H7A	0.3539	0.2136	0.0754	0.084*	
H7B	0.4450	0.3176	−0.0543	0.084*	
H7C	0.4384	0.3461	0.0788	0.084*	
C8	0.8227 (5)	0.1820 (4)	0.0586 (3)	0.0480 (7)	
H8A	0.8213	0.2500	−0.0369	0.072*	
H8B	0.9434	0.0827	0.0854	0.072*	
H8C	0.8175	0.2484	0.1081	0.072*	
C9	0.6694 (4)	−0.0318 (3)	0.1589 (2)	0.0309 (5)	
H9	0.5524	−0.0563	0.1773	0.037*	

### Atomic displacement parameters (Å^2^)

**Table:**

	*U*^11^	*U*^22^	*U*^33^	*U*^12^	*U*^13^	*U*^23^
Co1	0.0189 (2)	0.0105 (2)	0.0213 (2)	−0.00437 (16)	0.00295 (15)	−0.00778 (16)
O4	0.0233 (8)	0.0124 (7)	0.0237 (7)	−0.0055 (6)	0.0032 (5)	−0.0082 (6)
O1W	0.0208 (7)	0.0153 (7)	0.0301 (8)	−0.0054 (6)	0.0030 (6)	−0.0126 (6)
O3	0.0273 (8)	0.0180 (7)	0.0221 (7)	−0.0078 (6)	0.0063 (6)	−0.0110 (6)
O2	0.0279 (8)	0.0168 (7)	0.0297 (8)	−0.0076 (6)	0.0065 (6)	−0.0151 (7)
O1	0.0227 (8)	0.0146 (8)	0.0375 (8)	−0.0059 (6)	0.0044 (6)	−0.0144 (6)
O5	0.0393 (10)	0.0179 (8)	0.0395 (9)	−0.0122 (7)	0.0049 (7)	−0.0051 (7)
N1	0.0172 (9)	0.0136 (8)	0.0209 (8)	−0.0043 (7)	0.0028 (6)	−0.0087 (7)
N2	0.0200 (8)	0.0098 (8)	0.0240 (8)	−0.0056 (7)	0.0010 (6)	−0.0063 (7)
N3	0.0419 (11)	0.0206 (9)	0.0236 (9)	−0.0106 (8)	0.0034 (8)	−0.0076 (7)
C6	0.0152 (9)	0.0167 (10)	0.0228 (10)	−0.0060 (8)	0.0005 (7)	−0.0086 (8)
C5	0.0161 (9)	0.0155 (10)	0.0218 (9)	−0.0049 (8)	0.0017 (7)	−0.0094 (8)
C4	0.0163 (9)	0.0147 (10)	0.0227 (9)	−0.0057 (8)	0.0003 (7)	−0.0091 (8)
C3	0.0152 (9)	0.0160 (10)	0.0303 (10)	−0.0045 (8)	−0.0002 (8)	−0.0130 (9)
C2	0.0172 (10)	0.0128 (10)	0.0243 (10)	−0.0054 (8)	−0.0001 (7)	−0.0074 (8)
C1	0.0299 (11)	0.0205 (11)	0.0245 (10)	−0.0105 (9)	0.0045 (8)	−0.0096 (9)
C7	0.0534 (15)	0.0351 (15)	0.0471 (15)	0.0034 (13)	0.0177 (13)	−0.0078 (12)
C8	0.0650 (16)	0.0353 (14)	0.0426 (14)	−0.0331 (13)	−0.0161 (13)	0.0028 (12)
C9	0.0366 (13)	0.0268 (12)	0.0336 (12)	−0.0164 (11)	0.0115 (10)	−0.0143 (10)

### Geometric parameters (Å, °)

**Table:**

Co1—O1W^i^	2.0895 (14)	N2—H2	0.8600
Co1—O1W	2.0895 (14)	N3—C9	1.319 (3)
Co1—N1^i^	2.0982 (16)	N3—C8	1.440 (3)
Co1—N1	2.0982 (16)	N3—C7	1.453 (3)
Co1—O4	2.1543 (14)	C6—C5	1.476 (3)
Co1—O4^i^	2.1543 (14)	C5—C4	1.374 (3)
O4—C6	1.240 (2)	C4—C3	1.486 (3)
O1W—H1W2	0.8500	C2—C1	1.482 (3)
O1W—H1W1	0.8500	C1—H1A	0.9600
O3—C6	1.287 (2)	C1—H1B	0.9600
O2—C3	1.282 (3)	C1—H1C	0.9600
O2—H2A	0.863 (10)	C7—H7A	0.9600
O1—C3	1.237 (2)	C7—H7B	0.9600
O5—C9	1.237 (3)	C7—H7C	0.9600
N1—C2	1.327 (3)	C8—H8A	0.9600
N1—C5	1.371 (2)	C8—H8B	0.9600
N2—C2	1.353 (3)	C8—H8C	0.9600
N2—C4	1.368 (3)	C9—H9	0.9300
			
O1W^i^—Co1—O1W	180.00 (8)	N1—C5—C6	117.57 (17)
O1W^i^—Co1—N1^i^	90.58 (6)	C4—C5—C6	132.78 (18)
O1W—Co1—N1^i^	89.42 (6)	N2—C4—C5	105.63 (17)
O1W^i^—Co1—N1	89.42 (6)	N2—C4—C3	123.03 (18)
O1W—Co1—N1	90.58 (6)	C5—C4—C3	131.33 (19)
N1^i^—Co1—N1	180.0	O1—C3—O2	124.18 (18)
O1W^i^—Co1—O4	90.84 (5)	O1—C3—C4	119.29 (19)
O1W—Co1—O4	89.16 (5)	O2—C3—C4	116.53 (17)
N1^i^—Co1—O4	101.17 (6)	N1—C2—N2	110.61 (17)
N1—Co1—O4	78.83 (6)	N1—C2—C1	125.33 (18)
O1W^i^—Co1—O4^i^	89.16 (5)	N2—C2—C1	124.06 (18)
O1W—Co1—O4^i^	90.84 (5)	C2—C1—H1A	109.5
N1^i^—Co1—O4^i^	78.83 (6)	C2—C1—H1B	109.5
N1—Co1—O4^i^	101.17 (6)	H1A—C1—H1B	109.5
O4—Co1—O4^i^	180.0	C2—C1—H1C	109.5
C6—O4—Co1	113.98 (12)	H1A—C1—H1C	109.5
Co1—O1W—H1W2	114.5	H1B—C1—H1C	109.5
Co1—O1W—H1W1	114.9	N3—C7—H7A	109.5
H1W2—O1W—H1W1	106.7	N3—C7—H7B	109.5
C3—O2—H2A	108.9 (18)	H7A—C7—H7B	109.5
C2—N1—C5	106.13 (15)	N3—C7—H7C	109.5
C2—N1—Co1	142.57 (14)	H7A—C7—H7C	109.5
C5—N1—Co1	111.29 (12)	H7B—C7—H7C	109.5
C2—N2—C4	107.99 (16)	N3—C8—H8A	109.5
C2—N2—H2	126.0	N3—C8—H8B	109.5
C4—N2—H2	126.0	H8A—C8—H8B	109.5
C9—N3—C8	121.9 (2)	N3—C8—H8C	109.5
C9—N3—C7	120.7 (2)	H8A—C8—H8C	109.5
C8—N3—C7	117.2 (2)	H8B—C8—H8C	109.5
O4—C6—O3	123.66 (18)	O5—C9—N3	124.9 (2)
O4—C6—C5	118.29 (17)	O5—C9—H9	117.5
O3—C6—C5	118.04 (17)	N3—C9—H9	117.5
N1—C5—C4	109.64 (17)		
			
O1W^i^—Co1—O4—C6	−87.79 (14)	O4—C6—C5—C4	−179.21 (19)
O1W—Co1—O4—C6	92.21 (14)	O3—C6—C5—C4	−0.4 (3)
N1^i^—Co1—O4—C6	−178.54 (13)	C2—N2—C4—C5	0.0 (2)
N1—Co1—O4—C6	1.46 (13)	C2—N2—C4—C3	178.91 (17)
O1W^i^—Co1—N1—C2	−89.6 (2)	N1—C5—C4—N2	0.0 (2)
O1W—Co1—N1—C2	90.4 (2)	C6—C5—C4—N2	178.5 (2)
O4—Co1—N1—C2	179.4 (2)	N1—C5—C4—C3	−178.80 (19)
O4^i^—Co1—N1—C2	−0.6 (2)	C6—C5—C4—C3	−0.3 (4)
O1W^i^—Co1—N1—C5	89.19 (13)	N2—C4—C3—O1	−0.1 (3)
O1W—Co1—N1—C5	−90.81 (13)	C5—C4—C3—O1	178.5 (2)
O4—Co1—N1—C5	−1.78 (12)	N2—C4—C3—O2	−179.72 (17)
O4^i^—Co1—N1—C5	178.22 (12)	C5—C4—C3—O2	−1.1 (3)
Co1—O4—C6—O3	−179.57 (15)	C5—N1—C2—N2	0.0 (2)
Co1—O4—C6—C5	−0.8 (2)	Co1—N1—C2—N2	178.85 (15)
C2—N1—C5—C4	0.0 (2)	C5—N1—C2—C1	−179.54 (18)
Co1—N1—C5—C4	−179.25 (13)	Co1—N1—C2—C1	−0.7 (4)
C2—N1—C5—C6	−178.74 (17)	C4—N2—C2—N1	0.0 (2)
Co1—N1—C5—C6	2.0 (2)	C4—N2—C2—C1	179.55 (18)
O4—C6—C5—N1	−0.8 (3)	C8—N3—C9—O5	−2.9 (4)
O3—C6—C5—N1	178.00 (16)	C7—N3—C9—O5	−178.2 (2)

Symmetry codes: (i) −*x*, −*y*+2, −*z*+1.

### Hydrogen-bond geometry (Å, °)

**Table:**

*D*—H···*A*	*D*—H	H···*A*	*D*···*A*	*D*—H···*A*
N2—H2···O5^ii^	0.86	1.83	2.670 (2)	166
O2—H2A···O3	0.86 (1)	1.59 (1)	2.452 (2)	173 (3)
O1W—H1W2···O1^iii^	0.85	1.91	2.7579 (19)	178
O1W—H1W1···O1^iv^	0.85	2.03	2.847 (2)	160

Symmetry codes: (ii) −*x*+1, −*y*, −*z*+1; (iii) −*x*+1, −*y*+1, −*z*+1; (iv) *x*, *y*+1, *z*.
